# IL-6 Plasma Levels Correlate With Cerebral Perfusion Deficits and Infarct Sizes in Stroke Patients Without Associated Infections

**DOI:** 10.3389/fneur.2019.00083

**Published:** 2019-02-15

**Authors:** Benjamin Hotter, Sarah Hoffmann, Lena Ulm, Christian Meisel, Jochen B. Fiebach, Andreas Meisel

**Affiliations:** ^1^Charité – Universitätsmedizin Berlin, Corporate Member of Freie Universität Berlin, Berlin Institute of Health, Humboldt-Universität zu Berlin, Berlin, Germany; ^2^Center for Stroke Research Berlin, NeuroCure Clinical Research Center and Department of Neurology, Charité University Hospital Berlin, Berlin, Germany; ^3^Centre for Clinical Research, University of Queensland, Herston, QLD, Australia; ^4^Department of Medical Immunology, Charité University Medicine & Labor Berlin - Charité Vivantes, Berlin, Germany

**Keywords:** stroke, biomarker, MRI, IL-6, neuroinflammation

## Abstract

**Introduction:** We aimed to investigate several blood-based biomarkers related to inflammation, immunity, and stress response in a cohort of patients without stroke-associated infections regarding their predictive abilities for functional outcome and explore whether they correlate with MRI markers, such as infarct size or location.

**Methods:** We combined the clinical and radiological data of patients participating in two observational acute stroke cohorts: the PREDICT and 1000Plus studies. The following blood-based biomarkers were measured in these patients: monocytic HLA-DR, IL-6, IL-8, IL-10, LBP, MRproANP, MRproADM, CTproET, Copeptin, and PCT. Multiparametric stroke MRI was performed including T2^*^, DWI, FLAIR, TOF-MRA, and perfusion imaging. Standard descriptive sum statistics were used to describe the sample. Associations were analyzed using Fischer's exact test, independent samples *t-*test and Spearmans correlation, where appropriate.

**Results:** Demographics and stroke characteristics were as follows: 94 patients without infections, mean age 68 years (SD 10.5), 32.2% of subjects were female, median NIHSS score at admission 3 (IQR 2–5), median mRS 3 months after stroke 1 (IQR 0–2), mean volume of DWI lesion at admission 5.7 ml (SD 12.8), mean FLAIR final infarct volume 10 ml (SD 14.9), cortical affection in 61% of infarctions. Acute DWI lesion volume on admission MRI was moderately correlated to admission/maximum IL-6 as well as maximum LBP. Extent of perfusion deficit and mismatch were moderately correlated to admission/maximum IL-6 levels. Final lesion volume on FLAIR was moderately correlated to admission IL-6 levels.

**Conclusion:** We found IL-6 to be associated with several parameters from acute stroke MRI (acute DWI lesion, perfusion deficit, final infarct size, and affection of cortex) in a cohort of patients not influenced by infections.

**Clinical Trial Registration:** www.ClinicalTrials.gov, identifiers NCT01079728 and NCT00715533

## Introduction

Stroke is globally recognized as a leading cause for mortality and adult disability. While the advent of intravenous thrombolysis and recanalization has significantly improved the outcome of stroke, a significant proportion of patients persist to suffer from deficits and disabilities. This is partly explained by the course of stroke itself, but also depending on neurological and medical complications (1–3). Prognosis and precise prediction of outcome remains challenging, especially during the hyperacute phase of the disease. While demographic and clinical characteristics, such as i.e., age and severity of acute clinical syndrome, allow for an educated guess (4), the accuracy of prediction is limited (5). For the post-acute phase of the disease, recent progress has been made in prediction of motor and cognitive recovery (6, 7).

Development of more accurate scores to predict outcome in the acute setting may benefit from the inclusion of biomarkers. While individual association of sanguine parameters with stroke characteristics and functional outcome has been reported, no single biomarker stands out in the field. Immune parameters including cytokines and other acute phase proteins are indicative to the risk of post-stroke infections but have also been associated to functional outcome after stroke (8).

We aimed to investigate a range of inflammation-, immunity-, and stress-related biomarkers in a cohort of patients without stroke-associated infections regarding their predictive abilities for functional outcome and explore whether they correlate with MRI markers, such as infarct size or location.

## Methods

**Figure 1:**
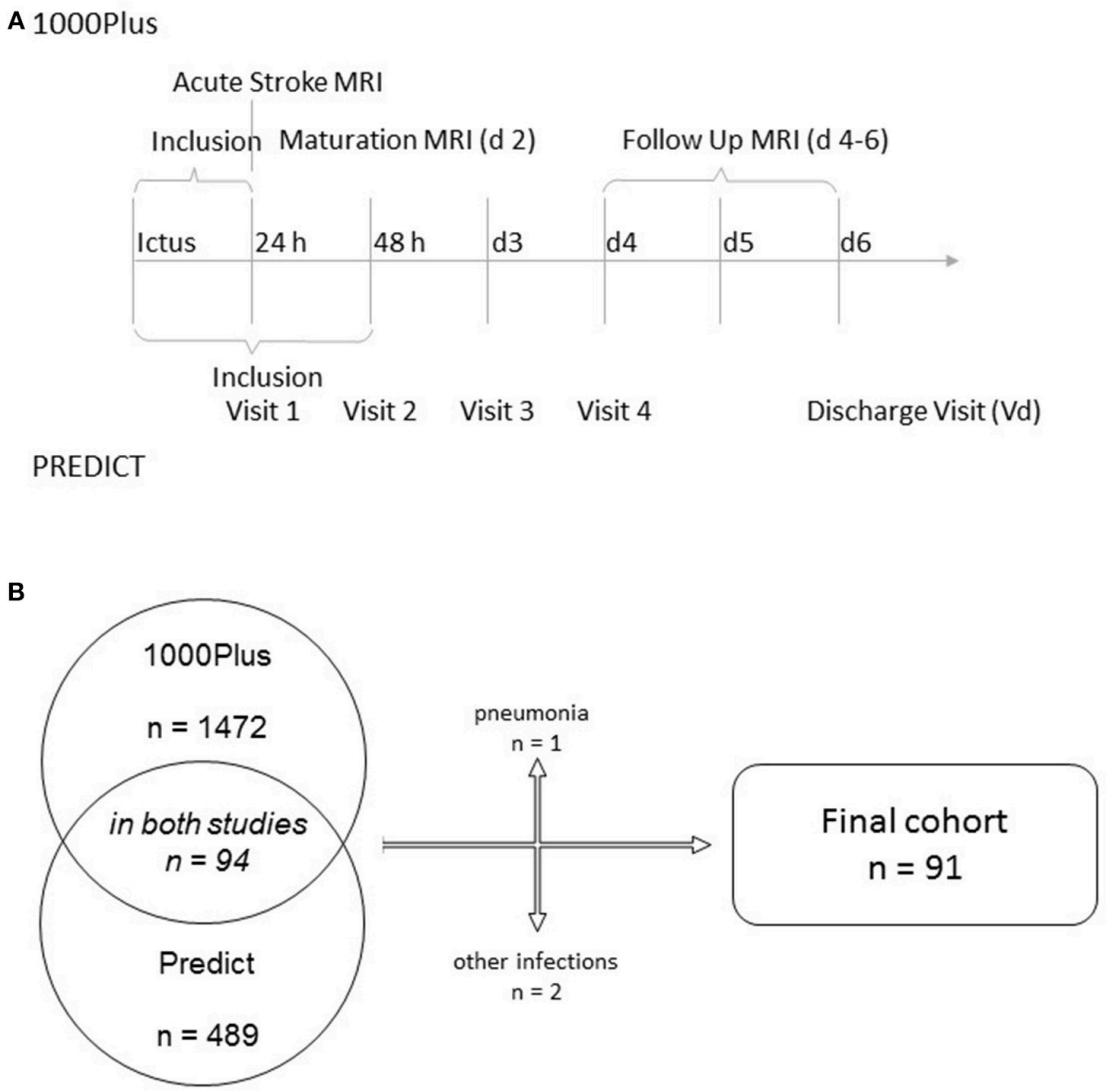
Timeline of the two studies supporting this analysis **(A)** and study flow chart **(B)**.

We combined the clinical and radiological data of patients participating in two observational acute stroke cohorts: the PREDICT and 1000Plus studies (clinicaltrials.gov NCT01079728 and NCT00715533). Both studies received full approval by the institutional ethics' committee, including the pooling of data for combined analysis. All patients (or if necessary, their legal representatives) gave informed consent to participation. Protocol details of both studies have been previously published (9, 10). Briefly, both studies recruited acute ischemic stroke patients to investigate either the prediction of stroke-associated pneumonia (PREDICT) or the natural course of multimodal MRI parameters (1000Plus), especially the evolution of DWI-perfusion imaging-mismatch. There was significant overlap of visit time points throughout the course of the study (see Figure 1). PREDICT was a multicentric study, with one of the participating centers (Charité Campus Benjamin Franklin) being the only study site in the 1000Plus study. PREDICT recruited 189 patients on that common site, 94 of which also participating in the 1000Plus study. We excluded patients suffering from an infection during their time of hospital admission for this analysis.

Blood samples parameters were obtained within the first 4 days of hospital admission. Samples were immediately post-processed and then frozen at −80°C in order to allow for batch analyses at the end of the study. Serum levels of mid-regional pro atrial natriuretic peptide (MRproANP), mid-regional pro adrenomedullin (MRproADM), C-terminal pro endothelin (CTproET), ultrasensitive copeptin (CPus), and ultrasensitive procalcitonin (PCTus) were measured using fluorescent immunoassays on the automated BRAHMS KRYPTOR compact PLUS™ analyzer (BRAHMS GmbH/Thermo Fisher Scientific, Henningsdorf, Germany) according to the manufacturer's protocol. The lower limits of quantitation were 4.5 pmol/l for MRproANP, 0.05 nmol/l for MRproADM, 3 pmol/l for CTproET, 1.9 pmol/l for CPus, and 0,02 μg/l for PCTus. Plasma concentrations of IL-6, IL-8, IL-10, and LBP were determined with the IMMULITE™ semi-automatic chemiluminescent immunoassay (Siemens Medical Solutions, Bad Nauheim, Germany). The detection limit for IL-6 and IL-8 is 2, 1 pg/ml for IL-10 and 0.8 μg/ml for LBP. Expression of human leukocyte antigen-DR (HLA-DR) on monocytes was determined in EDTA whole blood samples by flow cytometry using a highly standardized quantitative assay, as described earlier (9).

Multiparametric stroke MRI was performed on a 3T scanner (Tim Trio; Siemens AG, Erlangen, Germany) at admission (always within 24 h of the event), the following day and lastly 4–6 days after the event. Applied sequences contained T2^*^, DWI, FLAIR, TOF-MRA, and for the first two imaging time points also perfusion imaging. For further detail please refer to the published protocol (10). Admission MRI was performed as initial imaging upon presentation, or if outside of regular hours as the first examination the next morning. Follow-up MRIs were performed in the morning of the respective days. Blood samples were collected for the first day as soon as the patients and/or their legal representatives consented. The following samples were drawn with the routine laboratory rounds in the morning. Hence, delay between imaging and blood sampling was kept as short as logistically possible.

Standard descriptive sum statistics were used to describe demographics and stroke characteristics as well as biomarker and imaging results. Associations were analyzed by use of Fischer's exact test, independent samples *t-*test and Spearmans correlation, depending on character of variables. Alpha-error level was set at 2-tailed *p* = 0.05. All statistics were performed using SPSS (version 24.0, IBM, Armonk, NY, USA). In view of the small sample size and the explorative nature of the study, we decided against statistical correction such as Bonferroni.

## Results

We identified 94 patients participating in both Predict and 1000Plus. Three of them suffered from an infection during the course of their hospital stay and were excluded from this analysis (see Figure 1B for further detail). Mean age was 68 years (SD 10.5) and 32.2% of subjects were female. Median NIHSS score at admission was 3 (IQR 2–5). At 3 months, median mRS was 1 (IQR 0–2). Thrombolysis was applied in 23% of patients. For further details on clinical syndrome on admission, risk factors and stroke etiology refer to Table 1.

**Table 1:** Demographics and clinical stroke characteristics.

***n***	**91**
Sex, *n* (%) female	29 (32.2)
Age, years, mean (SD)	68.0 (10.5)
Admission NIHSS, median (IQR)	3 (2-5)
Admission mRS, median (IQR)	2 (1-3)
**ADMISSION SYMPTOMS**	
Aphasia [7]	10 (11.9)
Motor deficit	67 (77.9)
Dysarthria [7]	46 (54.8)
Dysphagia [11]	5 (6.3)
**RISK FACTORS**	
Diabetes mellitus	25 (28.7)
Atrial fibrillation	14 (16.1)
Previous stroke	16 (18.4)
Arterial hypertension	74 (85.1)
Hyperlipidemia	55 (63.2)
Smoking [61]	5 (16.1)
Thrombolysis	20 (23.0)
**TOAST**	
Large artery occlusion	56 (64.4)
Cardioembolism	18 (20.7)
Small artery disease	6 (6.9)
Other etiology	3 (3.4)
Unknown etiology	3 (3.4)
Concurring etiology	1 (1.1)
mRS 90 days after event, median (IQR) [17]	1 (0–2)

*Values given are n (%) if not explicitly stated otherwise, missing cases reported if >5 as [n]. NIHSS, National Institute of Health Stroke Scale; mRS, modified Rankin Scale; TOAST, Trial of Org10172 in Acute Stroke Treatment*.

Initial MRI was performed at a median of 549.5 min (IQR 130.25–860) after event. Mean volume of DWI lesion at admission was 5.7 ml (SD 12.8), mean final infarct volume as measured on FLAIR was 10 ml (SD 14.9). The right hemisphere was more frequently affected than the left hemisphere (52 vs. 37%) and cortex was involved in 61% of infarctions. Blood samples were collected during the first 4 days of hospitalization after stroke, and values for day of admission as well as maximum/minimum values are outlined in Table 2.

**Table 2:** Imaging and blood-based biomarker results.

**Hemisphere of DWI lesion**	
Right	46 (51.7)
Left	32 (36.0)
Bilateral	11 (12.4)
DWI lesion volume on acute MRI, ml	5.7 (12.8)
PI deficit volume on acute MRI, ml	45.1 (78.0)
DWI-PI mismatch on acute MRI, ml	26.4 (58.5)
**TYPE OF INFARCTION**	
Territorial	76 (85.4)
Lacunar infarction	12 (13.5)
Borderzone infarction	1 (1.1)
**REGIONS AFFECTED**	
Any Cortex	54 (60.7)
Caudate	3 (3.3)
Lenticulate	4 (4.4)
Capsula interna	8 (8.8)
Insula	11 (12.1)
ASPECT M1	6 (6.6)
ASPECT M2	17 (18.7)
ASPECT M3	14 (15.4)
ASPECT M4	15 (16.5)
ASPECT M5	34 (37.4)
ASPECT M6	21 (23.1)
FLAIR lesion volume on Follow Up, ml [33]	10.0 (14.9)
**HLA-DR, EPITOPES/CELL, MEDIAN (IQR)**	
At inclusion [3]	18,387 (14,434–25,788)
Lowest [1]	16,505 (11,386–21,285)
**IL-6, PG/ML, MEDIAN (IQR)**	
At inclusion [3]	2.8 (2.0–5.2)
Highest [1]	5.2 (2.7–11.0)
**IL-8, PG/ML, MEDIAN (IQR)**	
At inclusion [3]	5.0 (5.0–5.0)
Highest [1]	5.0 (5.0–5.2)
**IL-10, PG/ML, MEDIAN (IQR)**	
At inclusion [3]	5.0 (5.0–5.0)
Highest [1]	5.0 (5.0–5.0)
**LBP**, **μG/ML, MEDIAN (IQR)**	
At inclusion [3]	7.23 (5.48–9.34)
Highest [1]	8.90 (6.68–11.65)
**MPproANP, PG/ML, MEDIAN (IQR)**	
At inclusion [29]	111.4 (65.5–180.9)
Highest [23]	122.5 (79.1–195.8)
**MPproADM, PG/ML, MEDIAN (IQR)**	
At inclusion [29]	0.703 (0.593–0.868)
Highest [22]	0.751 (0.613–0.917)
**CTproET, PG/ML, MEDIAN (IQR)**	
At inclusion [30]	61.4 (50.7–75.9)
Highest [22]	64.4 (54.6–83.1)
**CPus, PG/ML, MEDIAN (IQR)**	
At inclusion [29]	8.84 (5.27–14.22)
Highest [22]	10.19 (5.60–17.40)
**PCT us, PG/ML, MEDIAN (IQR)**	
At inclusion [30]	0.031 (0.024–0.041)
Highest [23]	0.039 (0.028–0.050)

*Values given are n (%) if not explicitly stated otherwise, missing cases reported if >5 as [n]. HLA-DR, Human leukocyte antigen expression on monocytes; IL-6, IL-8, IL-10, three interleukins; LBP, lipopolysaccharide-binding protein; MRproANP, mid-regional pro atrial natriuretic peptide; MRproADM, mid-regional pro adrenomedullin; CTproET, C-terminal pro endothelin; CPus, ultrasensitive copeptin; PCTus, ultrasensitive procalcitonin*.

Age of patients was associated with IL-6, MRproANP, MRproADM, and CTproET. Stroke severity at admission as measured by National Institute of Health Stroke Scale (NIHSS) score was associated with IL-6. Functional outcome as measured by modified Rankin Scale (mRS) at day 90 was associated with admission levels of ultrasensitive Copeptin, maximum MRproADM, IL-6, and minimum HLA-DR levels (Table 3).

**Table 3:** Association of biomarkers at inclusion and lowest/highest measurement with clinical parameters.

		**Aphasia**	**Motor deficit**	**Dysarthria**	**Dysphagia**	**Sex**	**NIHSS**	**mRS d90**	**Age**
HLA-DR	Inclusion	0.405	0.979	0.548	0.757	0.606	0.409	0.052	0.249
	Lowest	0.987	0.436	0.959	0.173	0.340	0.099	**0.030**	0.069
IL-6	Inclusion	0.410	0.478	0.912	0.739	0.824	**0.005**	0.252	**0.001**
	Highest	0.369	0.281	0.983	0.438	0.884	**0.005**	**0.015**	**0.030**
IL-8	Inclusion	0.443	**0.050**	0.640	0.552	0.803	0.073	0.226	0.339
	Highest	0.633	0.393	0.386	0.467	0.597	0.331	0.683	0.566
IL-10	Inclusion	0.416	0.468	0.136	0.611	0.564	0.916	0.248	0.460
	Highest	0.247	0.360	0.257	0.727	0.989	0.683	0.587	0.236
LBP	Inclusion	0.086	0.364	0.958	0.660	0.227	0.291	0.952	0.148
	Highest	0.857	0.861	0.442	0.353	0.621	0.062	0.637	0.152
MPproANP	Inclusion	0.486	0.640	**0.012**	**0.029**	0.914	0.163	0.654	**0.005**
	Highest	0.521	0.287	**0.047**	**0.005**	0.926	0.068	0.911	**0.005**
MPproADM	Inclusion	**0.001**	0.320	0.310	0.915	0.296	0.241	0.264	**< 0.001**
	Highest	0.351	0.209	0.618	0.979	0.547	0.158	**0.043**	**0.001**
CTproET	Inclusion	0.398	0.323	0.613	0.994	0.962	0.160	0.344	**0.018**
	Highest	**0.015**	0.330	0.893	0.888	0.933	0.092	0.169	**0.039**
Copeptin us	Inclusion	0.720	0.455	0.548	0.436	0.357	0.768	**0.006**	0.540
	Highest	0.335	0.910	0.354	0.441	0.115	0.918	0.144	0.298
PCT us	Inclusion	0.643	0.381	**0.007**	0.736	0.286	0.254	0.962	0.975
	Highest	0.855	0.560	0.056	0.908	0.362	0.634	0.230	0.711

*Values given are two-sided p-values obtained by a students' t-test; Bold text denotes significant associations; HLA-DR, Human leukocyte antigen expression on monocytes; IL-6, IL-8, IL-10, three interleukins; LBP, lipopolysaccharide-binding protein; MRproANP, mid-regional pro atrial natriuretic peptide; MRproADM, mid-regional pro adrenomedullin; CTproET, C-terminal pro endothelin; CPus, ultrasensitive copeptin; PCTus, ultrasensitive procalcitonin*.

Acute volume of DWI restriction on admission MRI scans was moderately correlated to admission (Spearman's ρ 0.336) and maximum (Spearman's ρ 0.276) IL-6 as well as maximum LBP (Spearman's ρ 0.222) levels. Extent of perfusion imaging (PI) deficit and DWI-PI-Mismatch were moderately correlated to admission (Spearman's ρ 0.306 and 0.231, respectively) and maximum (Spearman's ρ 0.277 and 0.215, respectively) IL-6 levels. Final lesion volume on FLAIR was moderately correlated to admission IL-6 levels (Spearman's ρ 0.364) (Table 4). Cortical infarcts were associated with higher IL-6 levels at admission. By use of the ASPECT scoring system we only found inconsistent associations of biomarker levels with infarct location (Supplementary Table 1).

**Table 4:** Association of biomarkers at inclusion and lowest/highest measurement with imaging parameters.

		**Initial DWI volume**	**Initial PI deficit**	**Initial mismatch**	**Final FLAIR volume**
HLA-DR	At Inclusion	0.933	0.936	0.716	0.397
	Lowest	0.678	0.234	0.443	0.300
IL-6	At Inclusion	**0.002**	**0.016**	**0.030**	**0.006**
	Highest	**0.009**	**0.029**	**0.042**	0.383
IL-8	At Inclusion	0.955	0.667	0.928	0.068
	Highest	0.787	0.798	0.099	0.347
IL-10	At Inclusion	0.290	0.555	0.701	0.822
	Highest	0.081	0.101	0.595	0.435
LBP	At Inclusion	0.084	0.877	0.625	0.546
	Highest	**0.037**	0.349	0.431	0.688
MPproANP	At Inclusion	0.949	0.945	0.566	0.830
	Highest	0.853	0.795	0.486	0.941
MPproADM	At Inclusion	0.882	0.901	0.123	0.130
	Highest	0.682	0.801	0.267	0.340
CTproET	At Inclusion	0.923	0.887	0.665	0.827
	Highest	0.834	0.906	0.541	0.849
Copeptin us	At Inclusion	0.144	0.801	0.457	0.701
	Highest	0.487	0.673	0.648	0.815
PCT us	At Inclusion	0.804	0.214	0.224	0.637
	Highest	0.418	0.151	0.381	0.513

*Values given are two-sided p-values obtained by a Spearman's correlation; Bold text denotes significant associations; HLA-DR, Human leukocyte antigen expression on monocytes; IL-6, IL-8, IL-10, three interleukins; LBP, lipopolysaccharide-binding protein; MRproANP, mid-regional pro atrial natriuretic peptide; MRproADM, mid-regional pro adrenomedullin; CTproET, C-terminal pro endothelin; CPus, ultrasensitive copeptin; PCTus, ultrasensitive procalcitonin*.

## Discussion

We examined the relationship of inflammatory, immune, and stress biomarkers with MRI parameters in 91 stroke patients not suffering from stroke-associated infections during the course of the study. Interleukin-6 was associated with infarct size and tissue at risk, as well as final infarct volume. The other studied biomarkers did not show any associations with imaging markers in the absence of infection. Overall, the studied cohort was rather mildly affected by stroke (median admission NIHSS 3 IQR 2–5 and 3 months mRS 1 IQR 0–2).

The biological role of IL-6 in ischemic stroke remains uncertain. Astrocytes and microglia express IL-6, but whether it primarily exerts neurotoxic or—protective effects is a matter of scientific discourse (11, 12). Our study found a significant association of IL-6 levels with NIHSS scores at admission, although Spearman's ρ only showed moderate correlation. Furthermore, we found a significant correlation of IL-6 levels with lesion volume, whether on DWI scans at admission, PI deficit, DWI-PI mismatch or on follow-up FLAIR images, which is in line with the association of IL-6 levels and NIHSS scores at admission. Infarct size has previously been reported to correlate at least with intrathecal levels, but not consistently with serum levels, of IL-6 (13–15). IL-6 was repeatedly associated with poor functional outcome, but whether this is an independent effect or a signal due to infection remains unclear (8). Our data shows a significant association of IL-6 with functional outcome as measured by mRS in a cohort of patients not suffering from infection, further corroborating an association independent of infections. IL-6 has been previously linked to small vessel disease and silent cerebral infarctions (16–18). IL-6 and also IL-10 were associated with the presence of diffusion-perfusion- or clinical-diffusion-mismatch on acute stroke MRI (19–21). Furthermore, cortical infarcts were associated with IL-6. The associations of several other biomarkers with localization of lesions have to be interpreted with caution considering the sample size and plausibility.

Interestingly, in our cohort of patients not suffering from infections, plasma levels of IL-8 and IL-10 were mostly not detectable and below the upper limit of normal (5.0 pg/ml). This is in line with previous findings by us and Chamorro et al. showing increased IL-10 levels in patients with stroke-associated infections (22, 23). These cytokines may therefore be mainly triggered by systemic inflammation in the course of infectious complications after stroke, whereas neuroinflammation within the CNS does not seem to trigger their expression.

As previously described, we found functional outcome after 3 months to be inversely correlated to HLA-DR (24), and furthermore correlated to MRproADM and ultrasensitive Copeptin. Expression of monocytic HLA-DR is a marker of monocyte activation and has been shown to be a key marker for stroke-induced immune depression (9, 25). Lower HLA-DR expression is a strong predictor of stroke-associated pneumonia and is associated with worse functional outcome (9, 24). The latter association is further corroborated in our data independent of infection.

Several biomarkers showed an association with age, and while this has previously been reported for CTproET (26) and appears plausible for a vascular stress marker as MRproANP, the relationship for MRproADM and IL-6 is less seemingly obvious. LBP is a marker of bacterial translocation and higher levels are found in patients with post-stroke infection (27). It is also associated with a worse short-term stroke outcome (28). We could not reproduce this finding in stroke patients not suffering from infection or show any association with imaging characteristics. MRproANP is used as a biomarker for hemodynamic stress and was previously shown to indicate higher risk for ischemic stroke (29). There were no significant associations with imaging characteristics or functional outcome in our cohort. MRproADM exerts vasodilating, vasoprotective, and angiogenic effects and is associated with post-stroke infections (30) and functional outcome after stroke (31). MRproADM has been associated with progression of small vessel disease accompanying cognitive decline (32). We could not reproduce the association with poor outcome or find an association with imaging characteristics. The vasopressin surrogate CP has been associated with higher risk of all-cause mortality, poor functional outcome and infections after ischemic stroke (33–36). Furthermore, it has been proposed to improve prediction of recurring cerebrovascular events (37). CTproET is influenced by age, renal function, and hemodynamic parameters of healthy subjects, and is a strong vasoconstrictor (26). While it has not been studied as biomarker in ischemic stroke before, its derivative endothelin has been associated with carotid atherosclerosis and silent cerebral infarctions (38). Both biomarkers were not associated with outcome in our cohort, and did not show any significant correlations with imaging findings. PCT is a blood-based marker for infection in general and was shown to be associated with post-stroke infections and functional outcome (36, 39, 40). Furthermore, higher levels of PCT are associated with extent of small vessel disease and silent infarctions on MRI (41). Our data did not show an association with functional outcome or imaging characteristics.

Our study suffers from several limitations: the sample size, while average for a study in this field, is limited, especially considering the amount of analyses performed. While multiple testing was a concern for us, the purpose of this study was purely exploratory. Our findings need to be corroborated by further confirmatory studies. Furthermore, our study cohort was overall rather mildly affected by stroke, not necessarily reflecting a cohort of severely impaired patients. Strengths of this report are the prospective collection of data with an in-depth clinical and neuroradiological assessment during the acute course of the disease avoiding recall H bias.

Our data supports the conclusion that IL-6 is an inflammatory marker of cerebral parenchymal damage independent of systemic infections.

## Data Availability

The raw data supporting the conclusions of this manuscript will be made available by the authors, without undue reservation, to any qualified researcher.

## Author Contributions

BH wrote the manuscript and conducted all statistical analyses. SH and LU co-designed the PREDICT study and revised the manuscript. JF designed the 1000Plus study and was a major contributor in writing the manuscript. CM was a major contributor in writing the manuscript. AM designed the trial and was a major contributor in writing and revising the manuscript. All authors read and approved the final manuscript.

### Conflict of Interest Statement

The authors declare that the research was conducted in the absence of any commercial or financial relationships that could be construed as a potential conflict of interest.
