# Supplementary material for: IL-6 Plasma Levels Correlate With Cerebral Perfusion Deficits and Infarct Sizes in Stroke Patients Without Associated Infections
Source: Front Neurol. 2019 Feb 15;10:83. doi: 10.3389/fneur.2019.00083 (PMC6384225; doi:10.3389/fneur.2019.00083)
Supplement: Supplementary file 1 [file Table_1.DOCX]

Supplementary Material

Correlation of immunological biomarkers and MR imaging in acute ischemic stroke

Benjamin Hotter, MD*, Sarah Hoffmann, MD, Lena Ulm, MD, Christian Meisel, MD, Jochen B. Fiebach, MD, Andreas Meisel, MD

*** Correspondence:** Benjamin Hotter: benjamin.hotter@charite.de

# Supplementary Table 1

| Supplemental Table 1: Association of biomarkers at inclusion and lowest/highest measurement with location of infarction | | | | | | | | | | | |  |
| --- | --- | --- | --- | --- | --- | --- | --- | --- | --- | --- | --- | --- |
|  |  | ASPECT score items | | | | | | | | | | |
|  |  | Caudate | Lenticulate | Capsula interna | Insula | M1 | M2 | M3 | M4 | M5 | M6 | |
| HLA-DR | At Inclusion | 0.528 | 0.969 | 0.819 | 0.077 | 0.234 | 0.965 | 0.549 | 0.947 | 0.239 | 0.450 | |
|  | Lowest | 0.944 | 0.943 | 0.703 | 0.912 | 0.087 | 0.728 | 0.428 | 0.297 | 0.449 | 0.485 | |
| IL-6 | At Inclusion | 0.400 | 0.448 | 0.643 | 0.793 | 0.315 | 0.192 | 0.448 | 0.659 | 0.113 | 0.186 | |
|  | Highest | 0.439 | 0.960 | 0.487 | 0.494 | 0.937 | 0.381 | 0.332 | 0.982 | 0.093 | 0.747 | |
| IL-8 | At Inclusion | 0.651 | 0.600 | 0.500 | 0.416 | 0.555 | **0.017** | 0.420 | **0.017** | 0.682 | 0.286 | |
|  | Highest | 0.592 | 0.534 | 0.493 | 0.281 | 0.469 | 0.472 | 0.696 | **0.011** | 0.524 | 0.249 | |
| IL-10 | At Inclusion | 0.714 | 0.670 | 0.763 | 0.721 | 0.632 | 0.460 | 0.306 | 0.653 | 0.396 | 0.343 | |
|  | Highest | 0.625 | 0.290 | 0.595 | 0.608 | 0.680 | 0.384 | 0.384 | 0.537 | 0.939 | 0.547 | |
| LBP | At Inclusion | 0.753 | 0.096 | 0.396 | 0.288 | 0.882 | 0.442 | 0.430 | 0.729 | 0.884 | 0.700 | |
|  | Highest | 0.554 | 0.384 | 0.962 | 0.082 | 0.554 | 0.778 | 0.946 | 0.968 | 0.995 | 0.591 | |
| MPproANP | At Inclusion | **<0.001** | 0.427 | 0.336 | 0.138 | **0.013** | 0.084 | 0.236 | 0.204 | 0.425 | 0.453 | |
|  | Highest | 0.329 | 0.376 | 0.472 | 0.110 | **0.018** | 0.120 | 0.335 | 0.214 | 0.184 | 0.530 | |
| MPproADM | At Inclusion | 0.906 | 0.754 | 0.561 | 0.515 | 0.644 | 0.469 | 0.942 | 0.251 | 0.622 | 0.952 | |
|  | Highest | 0.679 | 0.529 | 0.640 | 0.908 | 0.274 | 0.905 | 0.666 | 0.494 | 0.850 | 0.469 | |
| CTproET | At Inclusion | 0.558 | 0.283 | 0.673 | 0.691 | 0.757 | 0.151 | 0.827 | 0.173 | 0.669 | 0.271 | |
|  | Highest | 0.835 | 0.192 | 0.424 | 0.470 | 0.419 | 0.092 | 0.753 | 0.154 | 0.849 | 0.339 | |
| Copeptin us | At Inclusion | 0.549 | 0.068 | 0.092 | 0.667 | 0.215 | 0.830 | 0.167 | 0.857 | 0.921 | **0.009** | |
|  | Highest | 0.739 | 0.180 | 0.160 | 0.620 | 0.431 | 0.598 | 0.224 | 0.716 | 0.731 | **0.005** | |
| PCT us | At Inclusion | 0.496 | **0.033** | 0.204 | 0.563 | 0.085 | 0.273 | 0.443 | 0.867 | 0.220 | 0.944 | |
|  | Highest | 0.759 | 0.107 | 0.213 | 0.706 | 0.211 | 0.531 | 0.760 | 0.862 | 0.545 | 0.787 | |
| Values given are two-sided p-values obtained by a students’ t-test; bold text denotes significant associations; Abbreviations: Human leukocyte antigen expression on monocytes (HLA-DR), three interleukins (IL-6, IL-8, IL-10), lipopolysaccharide-binding protein (LBP), mid-regional pro atrial natriuretic peptide (MRproANP), mid-regional pro adrenomedullin (MRproADM), C-terminal pro endothelin (CTproET), ultrasensitive copeptin (CPus) and ultrasensitive procalcitonin (PCTus) | | | | | | | | | | | |  |
